# The Management of Acute Anterior Uveitis Complicating Spondyloarthritis: Present and Future

**DOI:** 10.1155/2018/9460187

**Published:** 2018-10-14

**Authors:** Martina Biggioggero, Chiara Crotti, Andrea Becciolini, Elisabetta Miserocchi, Ennio Giulio Favalli

**Affiliations:** ^1^Department of Rheumatology, Gaetano Pini Institute, Milan, Italy; ^2^Department of Clinical Sciences and Health Community, University of Milan, Division of Rheumatology, Gaetano Pini Institute, Milan, Italy; ^3^Department of Ophthalmology, Scientific Institute San Raffaele, Milan, Italy

## Abstract

Spondyloarthropathies (SpA) encompass a group of chronic inflammatory diseases sharing common genetic and clinical features, including the association with HLA-B27 antigen, the involvement of both the axial and the peripheral skeleton, the presence of dactylitis, enthesitis, and typical extra-articular manifestations such as psoriasis, inflammatory bowel disease, and acute anterior uveitis (AAU). The latter is commonly reported as a noninfectious acute inflammation of the anterior uveal tract and its adjacent structures. AAU may affect more than 20% of SpA patients representing the most common extra-articular manifestation of the disease. Considering the potential consequences of untreated AAU, early diagnosis and aggressive treatment are crucial to avoid complications of remittent or chronic eye inflammation, such as visual loss and blindness. The management of SpA has dramatically improved over the last decades due to the development of new treat-to-target strategies and to the introduction of biologic disease modifying antirheumatic drugs (bDMARDs), particularly tumor necrosis factor alpha inhibitors (TNFis), currently used for the treatment of nonresponder patients to conventional synthetic agents. Along with the improvement of musculoskeletal features of SpA, bDMARDs provided an additional effect also in the management of AAU in those patients who are failures to topical and systemic conventional therapies. Nowadays, five TNFis, one interleukin-17, and one interleukin 12/23 blocker are licensed for the treatment of SpA, with different proven efficacy in preventing and treating ocular involvement. The aim of this review is to summarize the current options and to analyze the future perspectives for the management of SpA-associated AAU.

## 1. Introduction

Spondyloarthropathies (SpA) embrace different chronic inflammatory diseases sharing common genetic (association with HLA-B27 antigen) and clinical features. The principal symptoms are inflammatory chronic back pain, peripheral arthritis (typically asymmetric monoarthritis or oligoarthritis predominantly affecting the joints of the lower extremities), dactylitis, and enthesitis [1]. The disease course is usually complicated by extra-articular manifestations (EAMs), such as psoriasis, inflammatory bowel disease (IBD), and acute anterior uveitis (AAU) [2]. The latter is commonly reported as a noninfectious acute inflammation of the anterior uveal tract and its adjacent structures, which may affect more than 20% of SpA patients representing the most common EAM of the disease [3]. Considering the potential consequences of untreated AAU, early diagnosis and aggressive treatment are crucial to avoid the complications of remittent or chronic eye inflammation such as visual loss and blindness.

In this contest of very heterogeneous disease phenotype, the importance of personalised multidisciplinary management of the disease is mandatory. In the last decades, the development of new classification criteria allowing an earlier diagnosis and the availability of biologic and targeted synthetic therapies has vastly improved the management of SpA patients. Among biologic disease modifying antirheumatic drugs (bDMARDs), tumor necrosis factor alpha inhibitors (TNFis) are currently widely used for the treatment of SpA. To date, five TNFis (infliximab, adalimumab, etanercept, golimumab, and certolizumab pegol) have been licensed for SpA by the European Medicines Agency and the US Food and Drug Administration. Recently, new potential treatment targets in SpA emerged enhancing the available treatment options with novel mechanisms of action. In particular, blockers of interleukin-17 (IL-17; secukinumab and ixekizumab), interleukin 12/23 (IL-12/23; ustekinumab), and phosphodiesterase-4 (PDE4; apremilast) were included in the therapeutic armamentarium for SpA.

Although the efficacy and safety profiles of the different available therapies have been clearly demonstrated for the management of musculoskeletal features of SpA, different performances in preventing and treating ocular involvement were proven.

The aim of this review is to summarize the current options and to analyze the future perspectives for the management of SpA-associated AAU.

## 2. Classification of Uveitis and Epidemiology of AAU

Uveitis is one of the most common causes of blindness and represent a broad spectrum of disorders characterized by inflammation of the uveal tract (iris, ciliary body, and choroid) and its adjacent structures (vitreous humour, retina, optic nerve, and vessels). According to the Standardization of Uveitis Nomenclature (SUN) criteria, uveitis can be classified according to the anatomic site of inflammation into anterior (characterized by the presence of intraocular inflammation in the anterior chamber), intermediate (inflammation of the pars plana), posterior (inflammation of the posterior segment), or panuveitis (involving anterior and posterior segment) [14]. Uveitis can also be clinically classified by etiology as infectious (bacterial, viral, fungal, or parasitic), noninfectious (with known or unknown systemic association), and masquerade (heterogeneous group of eye diseases that mimic chronic intraocular inflammation). Noninfectious uveitis may be associated with many systemic autoimmune conditions or may occur without extraocular involvement (Table 1) [14]. Accurate data on prevalence of uveitis are lacking, because of differences in clinical and methodological case finding methods, but the reported annual incidence of uveitis is between 17 and 52 per 100.000 persons and the prevalence is 38-714 per 100.000 persons, despite the variability among different geographic areas worldwide [15]. Uveitis can occur at any age, but this disease more commonly affects the working population between 20 and 59 years. No prevalence variations are observed according to gender, but some forms present a sex predominance (i.e., juvenile idiopathic arthritis-related uveitis, more common in female, and HLA-B27 associated uveitis, more common in male) [16, 17]. As shown by epidemiological data, incidence differs among ethnicities: posterior uveitis and panuveitis are, respectively, the second and third most frequent locations in the Western countries (21% and 7%, respectively) [18]; this distribution may suggest a potential role played by genetic factors.

Anterior uveitis is the most common type of uveitis encountered in Western countries, while posterior and panuveitis are more frequently seen in developing countries due to the higher incidence of infectious uveitis involving the posterior segment of the eye. As mentioned before, the link between SpA and uveitis has been well described, since uveitis is the most common EAM in SpA and its main clinical presentation is with acute onset. However, about 50% of patients tend to have recurrent disease. [19]. It has been shown that AAU is the most common SpA-related type of uveitis accounting for almost 85% of cases in the USA [20]. A study conducted on more than 500 Spanish patients referred to an ophthalmologic centre for AAU reported that SpA was the most frequent systemic disease associated with AAU, diagnosed in about one quarter of cases [21]. In the DUET study, the prevalence of presumed idiopathic AAU, which was found to be associated with a SpA, was about 40% of patients [22]. The authors proposed an algorithm for early referral from ophthalmologists, in order to promptly diagnose an underlying SpA in presumed idiopathic AAU. Furthermore, HLA-B27 uveitis is commonly a nongranulomatous AAU [23]; so far, the lifetime cumulative incidence of AAU is higher in HLA-B27 positive subjects compared to general population, 1% versus 0.2%, respectively [24]. In the DUET study, HLA-B27 demonstrated to be the strong predictor of underlying SpA; in fact on multiple regression the detection of HLA-B27 was associated with an Odd's Ratio of 38.6. They suggest combining HLA-B27 positivity with low back pain to significantly improve the probability of an early diagnosis (sensitivity 95%, specificity 98%, and Likelihood Ratio 56 in the DUET algorithm) [22]. According to a systematic meta-analysis, the prevalence of AAU in SpA is 32.7% [19]. Prevalence enhances with disease duration, reaching 43% for over 30 years of disease [19], and varies between different forms: it is lower in undifferentiated SpA (13%) while it is higher in ankylosing spondylitis (AS) (33.2%) [19]. As shown by Zeboulon and colleagues [19], prevalence changes also according to the sex of patient: female prevalence is higher than male (Odds Ratio [OR] 1.3; confidence interval [CI] 95% 1,1-1,4). However, data on sex differences in prevalence of AAU have not been fully elucidated in the literature [25–27].

## 3. Pathogenesis of AAU in SpA

Definitive data about pathogenesis of AAU in SpA are still lacking, albeit some reports derived from experimental animal models have contributed to point out some evidences. Unlike other classical systemic inflammatory disorders, SpA are not characterized by defined serological markers to assist the diagnosis (e.g., autoantibodies), with the exception of HLA-B27 (a class I major histocompatibility complex–encoded allele). HLA-B27 is commonly linked to the whole group of diseases and in particular to AS [28] and has been included in the clinical arm of the classification criteria of axial SpA provided by the Assessment of Spondyloarthritis International Society (ASAS) [29]. Moreover, HLA-B27 has been associated with the development of SpA-related AAU [30–32], which is significantly more common in HLA-B27 positive patients compared to the negative ones (27.7% versus 9.7%, respectively; p<0.05) [33]. From a pathogenic point of view, HLA-B27 is involved in the development of SpA together with a rich mixture of over 20 genes [34] and with environmental factors such as entheseal mechanical stress and gut microbiome [35, 36]. Despite the more recent advances, our understanding of the molecular mechanisms of HLA-B27 in the pathogenesis of SpA and in particular SpA-related AAU is far from being complete. Models of HLA-B27 transgenic rats and mice develop spontaneous inflammatory diseases in gastrointestinal tract, vertebral joints, skin, and nails, but uveitis is infrequent, suggesting the need of additional factors in the induction of AAU [37, 38]. Another significant association between AAU and SpA was observed for other three nonmajor histocompatibility complex loci: IL23R, the intergenic region 2p15, and ERAP1 [39]. Besides the genetic component, the development of AAU involves other factors. The intravitreal injection of Gram-negative endotoxin can induce a bilateral, dose-dependent, self-limited AAU [40]. Moreover, recurrent uveitis similar to human AAU has been demonstrated in transgenic rats infected with Salmonella or Yersinia [41], suggesting the potential role for concomitant infections in determining the onset of full-blown AAU in individuals carrying a HLA-B27 genetic susceptibility to the disease. Finally, TNF alpha levels were observed to be high in both aqueous humor and serum of patients affected by noninfectious uveitis, with a direct correlation with the disease activity [42].

## 4. Clinical Presentation of AAU

The most common ocular symptoms of AAU are acute eye pain, redness, and intense photophobia. Nonspecific visual changes such as floaters and different degrees of visual acuity loss may be present [43]. AAU associated with SpA is frequently characterized by sudden onset and is often unilateral or unilateral alternating, anterior and recurrent.

Anterior uveitis associated with SpA is typically a nongranulomatous type of uveitis characterized by the presence of fine keratic precipitates visible at the slit lamp examination of the anterior segment. Intraocular pressure is usually low due to severe inflammation of the ciliary body. In severe forms of acute anterior uveitis, hypopyon and fibrin can be visualized as a white and dense clot in the anterior chamber.

Posterior synechia, cataract, and secondary glaucoma are the most common ocular complications of uveitis. In about 15 to 20 % of patients the uveitis may have a more severe and chronic course and may involve the posterior segment with macular edema, retinal vasculitis, and papillitis leading to visual loss [44].

Some authors speculate on the prognosis of HLA-B27 related AAU, reporting a higher frequency of recurrence and a worse outcome compared with HLA-B27 negative patients [45, 46].

The use of bDMARDs could significantly affect the prognosis of AAU associated with SpA [47]. In fact, the clinical course and the number of relapses of AAU have been hugely improved by TNFis [48]. Furthermore, the overall prognosis of AAU is quite good in TNFis treated SpA and only a minority of patients reported permanent visual loss [49].

## 5. Treatment of AAU in SPA

The management of uveitis reserves actually several clinical challenges. The therapeutic approach for uveitis requires careful consideration regarding etiology, involved anatomic site, chronicity, prior medication failure, and potential ophthalmic and systemic risks of proposed therapy: a definite diagnosis is crucial to establish an appropriate therapy. Treatment of noninfectious uveitis may be local, systemic, or a combination of the two. The therapeutic strategy evaluates the underlying diagnosis, severity of the disease, laterality, and presence of comorbidities. The treatment of SpA associated AAU should include the management of acute attack and the prevention of recurrences.

### 5.1. Topical Therapy

The first-line symptomatic treatment of acute attack of AAU consists in a cycloplegic agent combined with corticosteroids, which may be administered systemically, topically, or by subconjunctival injection. Periocular prednisolone acetate and intraocular dexamethasone are the most commonly used local treatment in patients with HLA-B27 associated uveitis with posterior pole complications. In a minority of cases, subconjunctival corticosteroid injections may be needed, only when a marked anterior segment inflammation results in significant loss of vision [50]. In the SITE (Systemic Immunosuppressive Therapy for Eye Diseases) cohort periocular corticosteroids were found to be effective in reducing intraocular inflammation and improving visual acuity in AAU [51]. An exciting area of research sustained by recent advances in bioengineering is the development of intravitreal implants releasing corticosteroids or other compounds. These delivery devices may be classified into surgical nonbiodegradable and bioerodible implants, with different durations and safety profiles [38]. These novel approaches for the delivering of therapeutic substances derive from the need of developing local therapies characterized by faster effect on targeted tissues, avoiding undesirable systemic side effects. The available ocular implants have been mainly developed for releasing corticosteroids such as dexamethasone or fluocinolone acetonide [52]. Topical cycloplegics are often used in tandem with topical corticosteroids to break and to prevent the formation of posterior synechiae.

### 5.2. Conventional Synthetic DMARDs Disease-Modifying Antirheumatic Drugs (csDMARDs)

The key for the treatment of noninfectious uveitis relapsing or refractory to topic therapy is the control of systemic inflammation generated by the underlying autoimmune disease [53]. Systemic corticosteroids are often administered when topical treatment is inadequately effective, especially for bilateral uveitis. However, the prolonged use of moderate to high doses is significantly limited by serious side effects related to corticosteroid cumulative dose over time [54], leading to the potential introduction of an immunosuppressive agent as a corticosteroid-sparing therapy. The addition of methotrexate produced the resolution of noninfectious uveitis, despite corticosteroid withdrawal, in about 60% of patients within one year [55]. Furthermore, it decreases the frequency of AAU flares during the progressive tapering of systemic corticosteroids treatment [56]. Data on sulfasalazine for the treatment of AAU are very limited, with a single paper reporting a significant reduction in the number of AAU flares and improvement in visual acuity in SpA [56]. Similarly, leflunomide had only few anecdotal data regarding the treatment of uveitis [57]. Azathioprine is moderately effective in the treatment of noninfective uveitis, mainly the intermediate form of the disease [58]. The use of systemic cyclosporine for intermediate and posterior uveitis is well described by several papers showing a comparable efficacy with corticosteroids [59]. In particular, cyclosporine offered both a complete remission in more than 30% and a significant corticosteroid-sparing effect in at least 20% of treated patients [60]. However, the lack of data on the treatment of anterior uveitis and the overall unfavorable long-term safety profile, in terms of nephrotoxic effects and hypertension, are main limitations for the extensive use of cyclosporine for the management of SpA-related AAU. Other systemic immunomodulatory medications for uveitis are now very infrequently used because of their potential toxicity, particularly for alkylating agents, in consideration of the availability of more targeted therapies such as biologics.

In conclusion, systemic immunosuppressive drugs have a potential in the management of AAU, even if their use showed no proven efficacy in the treatment of axial and enthesopathic involvement of SpA, limiting the opportunity to treat SpA-related AAU and the underlying disease with the same drug.

### 5.3. Biologic and Targeted Synthetic DMARDs

To date SpA has fewer therapeutic options than rheumatoid arthritis and could exhibit heterogeneous therapeutic responses considering different site involvements. Given the complexity of SpA, a tailored management of the disease that includes targeted DMARDs (biologic and small molecules) is mandatory.

#### 5.3.1. Role of Targeted DMARDs in SpA

Biologic DMARDs are defined as manufactured therapies by recombinant DNA (deoxyribonucleic acid) technology and include bioengineered soluble receptors, monoclonal antibodies, Fab fragments, and cytokines that affect the expression of pro- and anti-inflammatory components of the immune system. To date the major class of bDMARDs employed in SpA care is the successful use of TNF blockade in persistently high disease activity despite conventional treatments [61]. TNFis can be divided into three categories: a fusion protein that forms unstable complexes with the TNF (etanercept), monoclonal antibodies recognizing and binding to TNF (infliximab, adalimumab, and golimumab), and a Fab' fragment of a monoclonal antibody coupled with polyethylene glycol (certolizumab pegol). TNFis demonstrated to be highly effective in targeting the different disease musculoskeletal manifestations and could ameliorate the disability and quality of life, acting on general symptoms such as fatigue. Long-term follow-up studies suggest a retention rate maintained for several years of treatment with an optimal safety profile [62]. Nevertheless, a significant proportion of patients showed an inadequate or poor response and others experienced drug-related adverse events. Consequently, alternative mechanisms of action (MoA) may be welcomed for these patients. The IL-23/IL-17 axis is strongly implicated in the pathogenesis of SpA and there is increased interest in the potential role of therapeutic strategies targeting this way. Secukinumab, a high-affinity, fully human monoclonal antibody that selectively inhibits IL-17A, showed a rapid-onset efficacy in treating SpA with a wide range of clinical benefits [63–69]. Ixekizumab, a monoclonal antibody that selectively targets interleukin-17A actually licensed for PsO, improved the signs and symptoms of patients with active PsA with a safety profile [70, 71]. Ustekinumab, an anti-IL-12/IL-23 monoclonal antibody, is safe and effective for patients with active PsA and AS [72–74]. Recently, the therapeutic armamentarium for PsA has been enriched with apremilast, a phosphodiesterase-4 inhibitor that demonstrated clinically meaningful sustained improvements with a good tolerance and safety profile [75–77].

#### 5.3.2. Role of Targeted DMARDs in AAU

To date, bDMARDs have been used off-label to treat AAU because none of these therapies has been approved yet for adults, despite that their clinical efficacy has been reported in an amount of clinical cases and case series [78].

TNF alpha is essential in the intraocular immune response and in the autoregulation of the physiologic apoptosis of ocular cells. Preclinical studies give several evidences that TNF blocking can be a possible therapeutic strategy in uveitis. In fact, TNFis switch the immune response towards a Th2 prevalent mechanism, decreasing also disease activity [79]. Experimental autoimmune uveitis models highlighted that TNF alpha is increased not only in the typical autoimmune uveitis inflammatory infiltrates, but also in some retinal cells [80]. Moreover, the production of TNF alpha is regulated by ocular resident cells, macrophages, and activated T cells [81], possibly influencing the disease course. TNF alfa has a pivotal role in the pathogenesis of uveal inflammation; firstly, it recruits leukocytes to the eye in the early phase of the disease, through chemokines production and promotion of leukocytes adhesion to vascular endothelium. Secondly, TNF alpha promotes maturation of dendritic cells, improving the ability to act as presenting cells to T cells. Thirdly, TNF alpha can directly activate macrophages and promote T cells-effector function. Lastly, as mentioned, TNF alpha leads to apoptosis, of both infiltrating cells and resident ocular cells [82]. In clinical studies TNF alpha directly causes tissue damage through reactive oxygen species, breaking down the blood-ocular barrier and promoting angiogenesis [83]. TNF alpha could be related to endothelial tissue by the upregulation of vascular endothelial growth factor, whose effect is linked to cystoid macular edema and choroidal neovascularization [84]. These evidences give support to the use of TNFis in clinical practice. In fact, TNFis are the most frequently reported biologic drugs for the treatment of uveitis (Table 2). As was mentioned above, TNF alpha levels are high in both aqueous humor and the serum of patients affected by noninfectious uveitis; moreover there is a direct correlation between TNF alpha levels and the disease activity [42].

Retrospective studies on TNFis have been focused on underlying systemic disease with associated uveitis and some prospective studies have been successfully completed [78]. Several studies suggest that monoclonal antibodies are more effective than soluble receptors for the treatment of uveitis. The most important real-life experience reported with infliximab and adalimumab showed a clinical remission in over 60% of treated patients [15]. Further studies are needed to clarify a controversial area that is the potential paradoxical role of TNFis as a cause of uveitis [85, 86].

In animal models infliximab has shown a good safety profile and efficacy in the treatment of uveitis and dry eye and in scarring healing on the eye's surface [87–89]. It is successfully used also in Behçet associated uveitis and in JIA associated forms [90]. Infliximab has shown to be effective also in uveitis associated with other systemic immune mediated conditions rather than SpA, such as, sarcoidosis or inflammatory bowel diseases [91]. In a prospective study conducted on 23 patients with various underlying etiologies of resistant uveitis, 78 % of patients on infliximab therapy reported a clinical success at week 10, as judged by a composite clinical end point combining visual acuity, control of intraocular inflammation, ability to taper concomitant therapy, and improvement of fluorescein angiography and/or optical coherence tomography [92]. In a retrospective study on recalcitrant uveitis treated with infliximab, 81.8% of the patients achieved clinical remission and only 58.3% required additional immunomodulatory medications [93]. The mechanism of action of infliximab is related to the neutralization of soluble and membrane-bound form of TNF, as explained by its rapid and effective action, inhibiting a broad range of TNF action, as mentioned above [83]. Controversial results were obtained with intravitreal injection of infliximab when systemic administration is not indicated. Initially, this new route of administration showed promising results with a significant visual acuity improvement and macular thickness reduction in patients with chronic noninfectious uveitis [94, 95], unfortunately not confirmed in subsequent studies, reporting electroretinographic abnormalities, severe panuveitis, and modest efficacy for the long-term control of uveitis [96–98]. Adalimumab has a number of publications supporting its efficacy and good safety profile for the treatment of uveitis; it has demonstrated good responses in SpA-associated uveitis and HLA-B27-associated uveitis [91]. Recently, three prospective multicenter open-label phase III trials (VISUAL I, II, and III) have been conducted to assess the effectiveness and safety of adalimumab versus placebo [99–101]. In the VISUAL I study, 217 active noninfectious intermediate uveitis, posterior uveitis, or panuveitis, despite prior prednisone treatment for 2 or more weeks, were randomly assigned in a 1:1 ratio to receive adalimumab (a loading dose of 80 mg followed by a dose of 40 mg every 2 weeks) or matched placebo. The median time to adalimumab failure was 24 weeks, with an early and sustained separation of the treatment-failure curves, indicating that patients receiving adalimumab were significantly less likely to have treatment failure than those who received placebo. The VISUAL II trial assessed that adalimumab versus placebo significantly lowered the risk of uveitic flare or loss of visual acuity upon corticosteroid withdrawal in 229 patients with inactive, noninfectious uveitis controlled by systemic corticosteroids. Treatment failure occurred more frequently in the placebo group compared with the adalimumab one (55% versus 39%), as the time to treatment failure was significantly improved in the adalimumab group compared with the placebo one (p=0,004). The rate of adverse events was similar between groups. The impact of adalimumab on immunosuppressant use in 371 patients with active or inactive noninfectious intermediate, posterior, or panuveitis was analysed in the VISUAL III study, in which the long-term treatment with adalimumab reflected a reduction in csDMARDs dose and dependence in both groups.

Recently, these evidences had led to Food and Drug Administration and European Medicines Agency approval of adalimumab for the management of noninfectious intermediate uveitis, posterior uveitis, and panuveitis in adults. Adalimumab is licensed for the treatment of pediatric chronic noninfectious AAU in patients from 2 years of age, who have had an inadequate response to conventional therapies or in whom conventional therapy is inappropriate.

Etanercept is a therapeutic option in SpA but its efficacy on uveitis is much debated [11, 48, 91, 102, 103]. Braun and colleagues reported a greater reduction in uveitis flares with infliximab compared with etanercept in AS [48]. Similar results were observed in a retrospective analysis on 2115 AS patients with a higher risk of new-onset uveitis in patients treated with etanercept compared with monoclonal antibodies (infliximab and adalimumab) [12]. Galor et al. [104] compared etanercept to infliximab for the treatment of a variety of inflammatory eye diseases including HLA-B27-associated uveitis, showing a greater efficacy of infliximab compared with etanercept in decreasing the number of uveitis recurrences (0 versus 59%, respectively). On the other hand, in a meta-analysis Migliore et al. compared TNFis versus placebo in the treatment of uveitis in AS patients reporting a positive efficacy of all TNFis, including etanercept [103]. Accordingly, Kim et al. described a similar rapid improvement of uveitis with a reduction of the number of flare-ups in patients treated with infliximab, adalimumab, or etanercept [11].

Golimumab and certolizumab pegol efficacy in the treatment of uveitis has been reported only in few case reports and small case series of heterogeneous subgroups of patients, including patient nonresponders to prior TNFi [7, 105–110]. Further data are needed to make any statement.

The ability of other mechanisms of action to manage uveitis in SpA is still under delineation. The involvement of IL-23-IL17 pathway and the consequent pivotal role of autoreactive T cells in the pathogenesis of noninfectious uveitis provide a rationale for treatment of AAU with IL-17 inhibitors [33]. However, secukinumab did not meet the primary efficacy end points as a therapy in uveitis not specifically SpA-related in three RCTs versus placebo but reported a beneficial effect in reducing concomitant csDMARDs use [111]. A prospective nonrandomized pilot study to investigate ustekinumab as a possible treatment for active intermediate uveitis, posterior uveitis, or panuveitis (STAR study) is now ongoing (ClinicalTrials.gov Identifier: NCT02911116). Apremilast was not studied in AAU SpA-related. Ocular involvement is actually under assessment in a phase 3 randomized double-blind study designed to evaluate the efficacy and safety of apremilast in active Behcet's disease (ClinicalTrials.gov Identifier: NCT02307513) [112].

## 6. Future Perspectives

Although targeted therapies have provided a larger armamentarium to treat uveitis, challenges remain. Among small molecules, tofacitinib is an oral inhibitor of Janus kinase (JAK) 1 and 3 that is under investigation for the treatment of AS [113] and PsA in patients previously not responder to csDMARDs [114] or to TNFis [115]. To date, no clinical trials evaluating the efficacy of JAK inhibitors in uveitis have been conducted. The immunomodulatory effect of topical ophthalmic tofacitinib has been evaluated in dry eye disease, with a reduction of conjunctival cell surface HLA-DR expression and tear levels of proinflammatory cytokines and inflammation markers after 8 weeks of treatment [116].

## 7. Conclusions

The management of SpA and related AAU is extremely complex due to the pleomorphic characteristics of these diseases. Multidisciplinary approach is mandatory to achieve the target of an early diagnosis and aggressive treatment, in order to prevent disease progression and damage. The treatment armamentarium of SpA has been considerably improved over the last decades due to the development of new targeted drugs that provided an additional effect also in the management of AAU. The first line of treatment in AAU remains a combination of topical corticosteroids and mydriatic agents, reserving systemic corticosteroids for patients with refractory and severe involvement. The introduction of corticosteroid-sparing csDMARDs is a therapeutic option. Among csDMARDs, methotrexate and cyclosporine reported the most solid data in AAU treatment with an acceptable safety profile. TNFis are the most frequently used bDMARDs in the treatment of both SpA and AAU. In particular, monoclonal antibodies TNFis resulted more effective than etanercept in AAU potentially due to the paradoxical effect and a lower efficacy of the fusion protein. New mechanisms of action targeting the IL-23-IL17 pathway are still under delineation and further data are needed to make any statement.

## Figures and Tables

**Table 1:** Autoimmune disorders associated with noninfectious uveitis.

Systemic immune-mediated causes of uveitis	Uveitis syndromes confined primarily to the eye
(i) Ankylosing spondylitis	(i) Acute posterior multifocal placoid pigmentary epitheliopathy
(ii) Behçet's disease	(ii) Acute retinal necrosis
(iii) Blau syndrome	(iii) Autosomal dominant neovascular inflammatory vitreoretinopathy
(iv) Crohn's disease	(iv) Birdshot choroidopathy
(v) Drug or hypersensitivity reaction	(v) Fuchs heterochromic cyclitis
(vi) Interstitial nephritis	(vi) Glaucomatocyclitic crisis
(vii) Juvenile idiopathic arthritis	(vii) Immune recovery uveitis
(viii) Kawasaki's disease	(viii) Iridocorneal endothelial syndrome
(ix) Multiple sclerosis	(ix) Leber's stellate neuroretinitis
(x) Neonatal onset multisystem inflammatory disease	(x) Multifocal evanescent white dot syndrome
(xi) Psoriatic arthritis	(xi) Pars planitis
(xii) Reactive arthritis	(xii) Punctate inner choroidopathy
(xiii) Relapsing polychondritis	(xiii) Serpiginous choroidopathy
(xiv) Sarcoidosis	(xiv) Subretinal fibrosis and uveitis syndrome
(xv) Sjögren's syndrome	(xv) Sympathetic ophthalmia
(xvi) Sweet syndrome	(xvi) Trauma
(xvii) Systemic lupus erythematosus	
(xviii) Ulcerative colitis	
(xix) Vasculitis	
(xx) Vitiligo	
(xxi) Vogt-Koyanagi-Harada syndrome	

**Table 2:** Characteristics of main trials on Spondyloarthritis-related anterior acute uveitis treated with biological DMARDs.

Reference	Study design	Diagnosis	Number of patients	Drug	Outcomes
Dobner BC 2013 [4]*∗*	Observational, retrospective, multicentric	SpA	60	ADA	Improvement criteria of visual activity and steroid sparing

van Denderen JC 2014 [5]	Observational, prospective	AS	71	ADA	Number of flares before and after drug treatment

Rudwaleit M 2009 [6]	Prospective open-label study	AS	274	ADA	Number of AAU flares

Hernandez MV 2016 [7]	Observational, multicentric, retrospective	SpA	14	CZP	Visual acuity

Yazgan S 2016 [8]	Observational, retrospective	AS	12	GOL	Steroid sparing, visual acuity and number of flares

Calvo-Río V 2016 [9]	Observational, prospective	SpA	15	GOL	Visual acuity

Faez S 2013 [10]	Retrospective case series	SpA	3	GOL	Visual acuity

Kim M 2016 [11] *∗∗*	Retrospective cohort study	AS	143	ADA, IFX, ETN	Number of flares and reduction in systemic medication

Wendling D 2014 [12]	Retrospective, cohort study	AS	2115	IFX, ADA, ETN	Risk of developing AAU

Lie E 2017 [13]	Observational, retrospective	AS	1365	IFX, ADA, ETN	AAU incidence before and after treatment

ADA: adalimumab; IFX: infliximab; ETN: etanercept; GOL: golimumab; CZP: certolizumab Pegol; AAU: acute anterior uveitis; SpA: Spondyloarthritis; AS: Ankylosing spondylitis.

*∗* In this study authors considered uveitis in general; however AAU were in 83.3% of patients.

*∗∗* In this study authors considered uveitis in general; however AAU were the majority of cases: 88% of patients in IFX group, 97% in ADA, and 63.2% in ETN.
